# Influenza virus exploits tunneling nanotubes for cell-to-cell spread

**DOI:** 10.1038/srep40360

**Published:** 2017-01-06

**Authors:** Amrita Kumar, Jin Hyang Kim, Priya Ranjan, Maureen G. Metcalfe, Weiping Cao, Margarita Mishina, Shivaprakash Gangappa, Zhu Guo, Edward S. Boyden, Sherif Zaki, Ian York, Adolfo García-Sastre, Michael Shaw, Suryaprakash Sambhara

**Affiliations:** 1 Immunology and Pathogenesis Branch, Influenza Division, Centers for Disease Control and Prevention, 1600 Clifton Road, Atlanta, GA 30329-4027, USA; 2 Infectious Diseases Pathology Branch, Division of High-Consequence Pathogens and Pathology, Centers for Disease Control and Prevention, 1600 Clifton Road, Atlanta, GA 30329-4027, USA; 3 Virus Surveillance and Diagnostics Branch, Influenza Division, Centers for Disease Control and Prevention, 1600 Clifton Road, Atlanta, GA 30329-4027, USA; 4 Media Lab, McGovern Institute, Department of Brain and Cognitive Sciences, MIT, Cambridge, MA, USA; 5 Department of Microbiology, Department of Infectious Disease, Global Health and Emerging Pathogens Institute and Department of Medicine, Division of Infectious Diseases, Icahn School of Medicine at Mount Sinai, New York, USA; 6 Office of Infectious Diseases, Centers for Disease Control and Prevention, 1600 Clifton Road, Atlanta, GA 30329-4027, USA

## Abstract

Tunneling nanotubes (TNTs) represent a novel route of intercellular communication. While previous work has shown that TNTs facilitate the exchange of viral or prion proteins from infected to naïve cells, it is not clear whether the viral genome is also transferred via this mechanism and further, whether transfer via this route can result in productive replication of the infectious agents in the recipient cell. Here we present evidence that lung epithelial cells are connected by TNTs, and in spite of the presence of neutralizing antibodies and an antiviral agent, Oseltamivir, influenza virus can exploit these networks to transfer viral proteins and genome from the infected to naïve cell, resulting in productive viral replication in the naïve cells. These observations indicate that influenza viruses can spread using these intercellular networks that connect epithelial cells, evading immune and antiviral defenses and provide an explanation for the incidence of influenza infections even in influenza-immune individuals and vaccine failures.

Influenza A virus (IAV) is a member of the Orthomyxoviridae family that contains a negative-strand segmented RNA genome and is notorious for its ability to evolve and evade immune responses. IAV enters the host cell via receptor-mediated endocytosis, replicates and newly synthesized viruses are released apically and/or basolaterally which infect the neighboring cells^1^. Neutralization of the invading virus with antibodies induced either by prior infection or vaccination is the primary mechanism to prevent influenza infection. However, despite the presence of circulating protective levels of hemaglutination inhibiting antibodies, influenza viruses can still spread to cause disease, the underlying mechanisms of which are not clear^2^. Therefore, we investigated the evasive strategies used by IAV in the presence of antibodies as well as antiviral agents.

Tunneling nanotubes (TNTs) are long membranous actin based extensions that connect one cell to another to allow exchange of cellular organelles and signaling molecules between two connected cells^3^,^4^,^5^,^6^,^7^. Previous work has shown that TNTs allow the exchange of human immunodeficiency virus-group specific antigen-green fluorescent protein (Gag-GFP) or GFP–tagged prion proteins from infected Jurkat or neuronal cells, respectively, to naïve cells^7^,^8^. Roberts *et al*.^9^ recently demonstrated the transfer of viral proteins via TNTs in a population of cells infected with influenza or parainfluenza virus 5^9^. However, it is not clear whether the viral genome is also transferred, and further whether transfer via this mechanism facilitates replication of the infectious agents in the recipient cell. Therefore, we set up an experimental system to selectively sort the uninfected naïve cells after co-incubating them with a population of infected cells in the presence of hemaglutination inhibiting antibodies and the antiviral drug, Oseltamivir, and examined the presence of IAV viral protein and/or RNA in the un-infected population. We anticipated that this experimental design would reveal the contribution of TNTs in facilitating cell-to-cell spread of IAV.

## Results and Discussion

### Lung Epithelial cells are connected by a network of tunneling nanotubes

**Figure 1:**
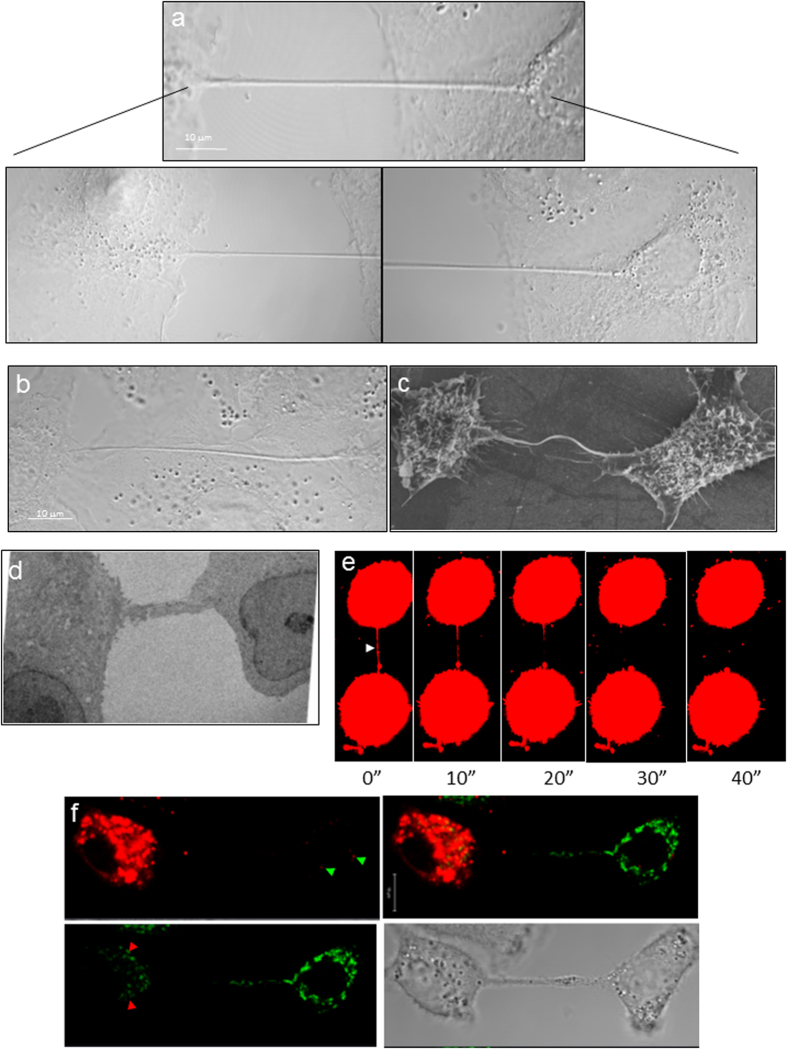
TNTs connect neighboring epithelial cells. (**a** and **b**) TNT connecting neighboring A549 cells in a non-confluent monolayer (**a**) or a confluent monolayer (**b**) as imaged using the method of expansion microscopy. Lower panels show a higher magnification of the TNT at the cellular ends of the TNT shown in the upper panel. The scale bar corresponds to 10 μm. For both panels a and b, samples were chemically fixed, infused with polyelectrolyte gel, followed by dialysis in water, and image captured with a Zeiss confocal microscope equipped with a 100x/1.4 NA oil objective. (**c**) Ultra structure of TNTs. Scanning electron microscopy (SEM) shows the ultrastructure of a TNT between two A549 cells. Scale bar corresponds to 6.6 μm. (**d**) A TEM section of A549 cells cultured for 4 h after seeding, indicates the presence of TNTs connecting neighboring A549 cells. (**e**) Selected frames of a video sequence showing the dynamic and transient nature of TNTs connecting two A549 cells. Arrowhead highlights a TNT connecting two cells that bursts 45 sec after exposure to high laser power. (**f**) Mitochondria transfer via TNTs connecting two A549 cells. A549 cells were stained with Mitotracker Green or Mitotracker Red, mixed 1:1, and co-incubated for an additional 4 hrs before imaging. Green arrowheads highlight a red-stained mitochondria in cells stained with Mitotracker Green and red arrowheads highlight a green-stained mitochondria in cell stained with Mitotracker Red.

Epithelial cell lines (A549 and MDCK) and primary bronchial epithelial cells (NHBE) support efficient replication and spread of IAV^10^,^11^,^12^. TNTs are known to occur naturally in several cell types^3^,^4^,^5^,^6^,^7^. To study TNT formation in lung epithelial cells, we trypsinized 60–80% confluent A549 cell culture, seeded them on glass-bottom culture dishes, and imaged within 2–4 h of seeding using 3D-live-cell microscopy and expansion microscopy^13^. Imaging through a fixed sample of A549 cells using the method of expansion microscopy showed that TNTs formed a continuous connection between two cells (Fig. 1a and b). Similar TNT connections could also be observed in live cell samples grown on glass bottom plates and imaged using live cell 3D microscopy (Supplementary Figure 1a and b). No differences in frequency of TNT formation were observed when cells were cultured in either low serum or complete media (data not shown), and only 8–10% of cells counted (n > 500) were connected by TNTs that ranged in size from 1–2 μm to extending as long as > 100 μm (Supplementary Figure 1c). In A549 cells, TNTs came in multiple forms such as curved, straight, single, multiple or branched (Supplementary Figure 1d–g). Reconstruction of the 3D-fluorescence images using depth analysis that projects the scanned image along the z-depth shows that these bridges lie several microns above the substratum (Supplementary Figure 2a, Supplementary movie 1), thus distinguishing them from filopodial bridges that connect cells^14^. Similar formation of TNTs was also observed in MDCK cells (Supplementary Figure 2b). These initial findings are consistent with earlier reports of TNTs in neuronal cells, immune cells, and epithelial cells^6^,^7^,^8^,^9^,^15^.

To establish that TNTs provide membrane continuity between the connected cells, we co-incubated a population of cells labelled with either membrane dye 1,1′-dioctadecyl-3,3,3′,3′-tetramethylindodicarbocyanine perchlorate (DiD, red) or 3,3′-dioctadecyloxacarbocyanine perchlorate (DiO, green). As shown in Supplementary Figure 2c, both red and green dye was observed within the TNT, and exchange of the dyes had occured as observed by red punctate staining in cells initially labelled with the green color and vice-versa. To examine the continuity at a higher resolution, TNTs were imaged with either a scanning electron microscope (SEM) or transmission electron microscopy (TEM) in A549 (Fig. 1c and d, respectively) and MDCK cells (Supplementary Figure 3a and b, respectively). While SEM images show TNT connecting two cells, electron micrographs revealed that the cellular ends of these bridges have continuous connection with the cytoplasmic contents of the cells (see panels i and v in Supplementary Figure 4). Analysis of more than 10 TNTs imaged in A549 cells by SEM showed that the average diameter of the TNTs as observed in A549 cells varied from 0.25–1.37 μm.

TNTs were easily labelled with phalloidin (Supplementary Figure 1d and Supplementary Figure 5a) or alpha-tubulin (Supplementary Figure 5a) and the results indicate that these cellular extensions in A549 and MDCK cells contain actin and tubulin. High resolution TEM scan of the TNTs, also show dense bundling of fibers that run along the length of the TNT (Supplementary Figure 4) suggesting that TNT’s consist of fibers. While, the presence of actin filaments has been described in multiple cell types, the presence of tubulin filaments within TNTs depends on cell type. The presence of tubulin in A549 cells is unlike that reported for TNTs of T lymphocytes^7^,^8^, or those reported by Roberts *et al*.^9^ and Wittig *et al*.^16^ in epithelial cells^9^,^16^. Differences in experimental approaches, timing, and the growth conditions of the cell-lines may be contributing to these observed differences.

TNTs formed dynamically (Supplementary Figure 5b and Supplementary movie 2) when two cells in close proximity moved apart over time (Supplementary movie 3). In this movie the formation of the TNT was seen to occur over a period of 4–5 h. Often TNTs were very fragile, and ruptured when exposed to high laser power ( > 60% laser power with the 561 nm laser) in the fluorescent confocal microscope (Fig. 1e, Supplementary movie 4). Similar breakage in TNTs was also observed when the electron beam intensity was increased during image capture with the SEM (Supplementary Figure 5c).

### Tunneling nanotubes facilitate the exchange of cellular organelles

Given that the diameter of the nanotubes as observed by SEM imaging varied from 0.25–1.37 μm, and studies have shown that TNT’s facilitate transfer of organelles, we examined whether the TNTs observed in A549 cells facilitate exchange of cellular organelles^9^,^16^,^17^. To examine the exchange of organelles, we stained A549 cells with either Mitotracker Red or Mitotracker Green, co-incubated the two differentially labelled populations, and imaged 2–4 h after seeding on a glass-bottom plate. Figure 1f shows the presence of mitochondria (green color) in the cytoplasm of cells whose mitochondria were stained with Mitotracker Red and vice versa. Further, TEM analysis shows the presence of many mitochondria and ribosomes in a TNT-like extension from a cell (Supplementary Figure 6) suggesting that these connections facilitate the exchange of cellular contents between connected cells.

### Tunneling nanotubes contain influenza virus proteins and genome

**Figure 2:**
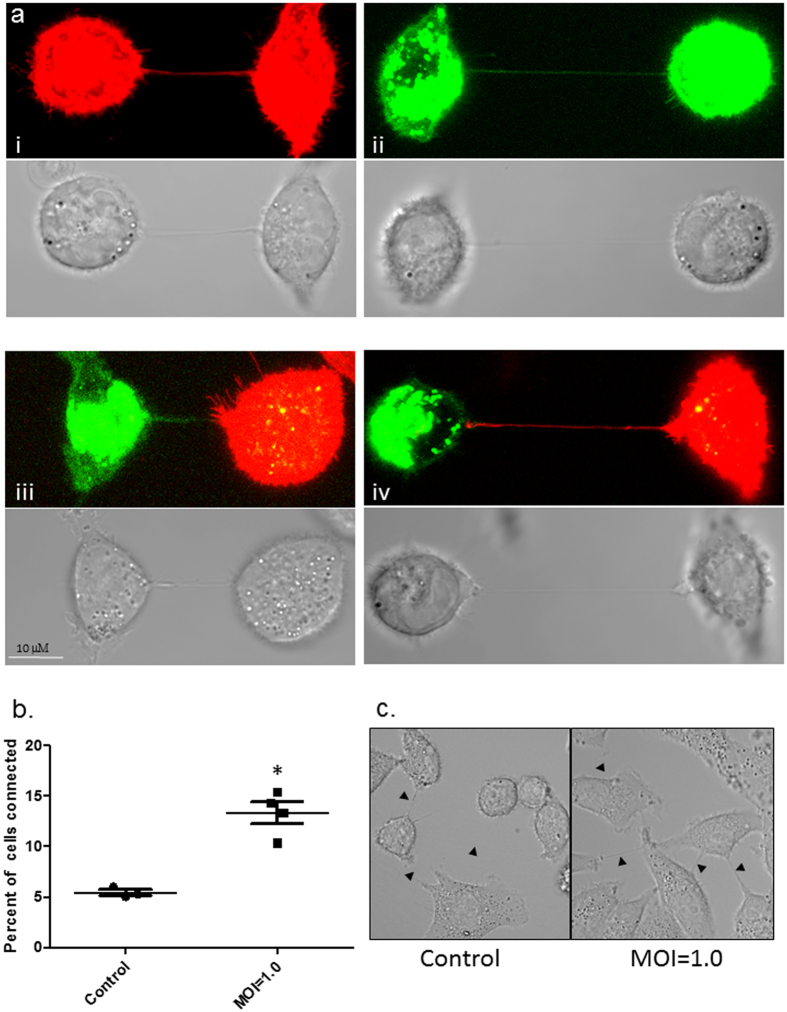
TNTs form between infected and uninfected A549 cells. (**a**, panel i and ii) TNTs readily form between uninfected A549 cells labelled with membrane dye DiD (red), or between A549 cells infected with PR8 virus and labelled with DiO (Green). After 1 h of co-culture nanotubes formed by uninfected and infected A549 cells were assessed (Scale bars: 10 μm). (**a,** panel iii and iv) TNTs were also seen readily connecting neighboring A549 cells either uninfected (red) or PR8 infected (green) cells. Corresponding bright field images are also shown. (**b** and **c**) Panel showing increased frequency of TNT formation in A549 cells infected with PR8 virus when compared to uninfected cells. The frequency of TNT formation between uninfected and PR8 infected A549 cells is shown after 2–4 h of incubation at 37 °C (n = 500; number of cell counts over three independent experiments); difference between uninfected and infected cells is significant (t-test, P < 0.05).

**Figure 3:**
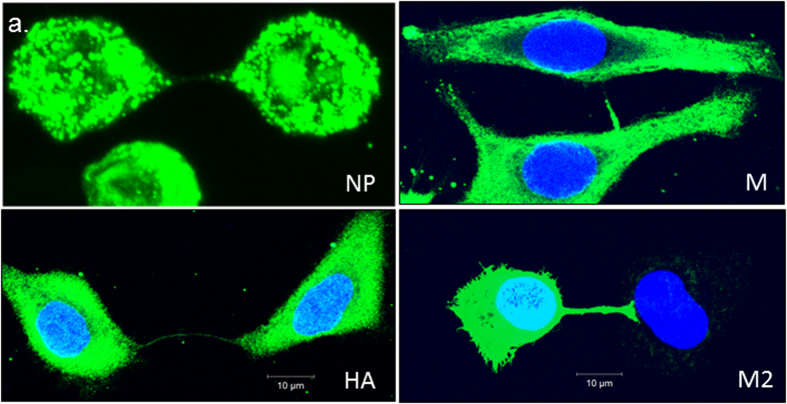
TNTs serve as a conduit for intracellular transport of IAV proteins. Fluorescent images showing accumulation of influenza virus proteins NP, HA, M, and M1 proteins within TNTs. A population of A549 cells was infected with PR8 virus for 18 h, trypsinized and seeded on glass-bottom plates for 2 h after which they were imaged as described in materials and methods. Viral proteins are observed in the membrane nanotubes.

Infection of many viruses, including influenza viruses, is facilitated by receptor-mediated endocytosis with viral progeny being released apically and/or basolaterally, allowing newly released progeny to infect neighboring cells^1^,^18^. In addition to these mechanism of viral spread, several papers describe that viral proteins including that of influenza virus can be transferred via TNTs^7^,^9^,^19^. However, it is not clear whether such connections exist between infected and uninfected cells and whether viruses can exploit these existing connections to spread to neighboring cells. To determine whether connections form between infected and uninfected cells, we labelled a population of cells infected with PR8 virus with DiO (green) and co-cultured them with naïve A549 cells stained with membrane dye DiD (red). Analysis 2–4 h after seeding showed both infected (green) or uninfected (red) cells were capable of forming TNTs (Fig. 2a, panel i–ii). Further, connections were observed between uninfected and infected cells, and both infected and uninfected cells had the ability to form the TNT as observed by TNTs of either green or red color (Fig. 2a, panel iii and iv). Infection with PR8 virus increased the frequency of nanotubes, suggesting that the virus may be utilizing these cellular process to spread (Fig. 2b and c). Increase in numbers of TNTs have also been observed in cells infected with parainfluenza virus 5 or IAV^9^. Similarly, for human immunodeficiency virus (HIV), Eugenin *et al*. observed an increase in TNT in infected macrophages^20^,^21^. However, Sowinski *et al*. did not observe a similar increase in Jurkat cells (T cells) infected with HIV^7^. To examine the presence and/or transfer of influenza viral proteins through TNTs, we next stained for the presence of IAV Hemagglutinin (HA), Nucleoprotein (NP), Matrix protein (M) and Matrix protein 2 (M2) in nanotubes of infected cells. After infection with PR8 virus for 18 h, NP, HA, M and M2 labeling was frequently detected in membrane nanotubes (Fig. 3 and Supplementary Figure 7). No staining was observed in antibody only control cells, or in cells stained with secondary antibody only (data not shown). These results confirm the observations of Roberts *et al*., who showed that IAV proteins are observed within these connections between cells^9^.

### Transfer of viral genome and protein via tunneling nanotubes results in establishment of productive infection in naïve cells

**Figure 4:**
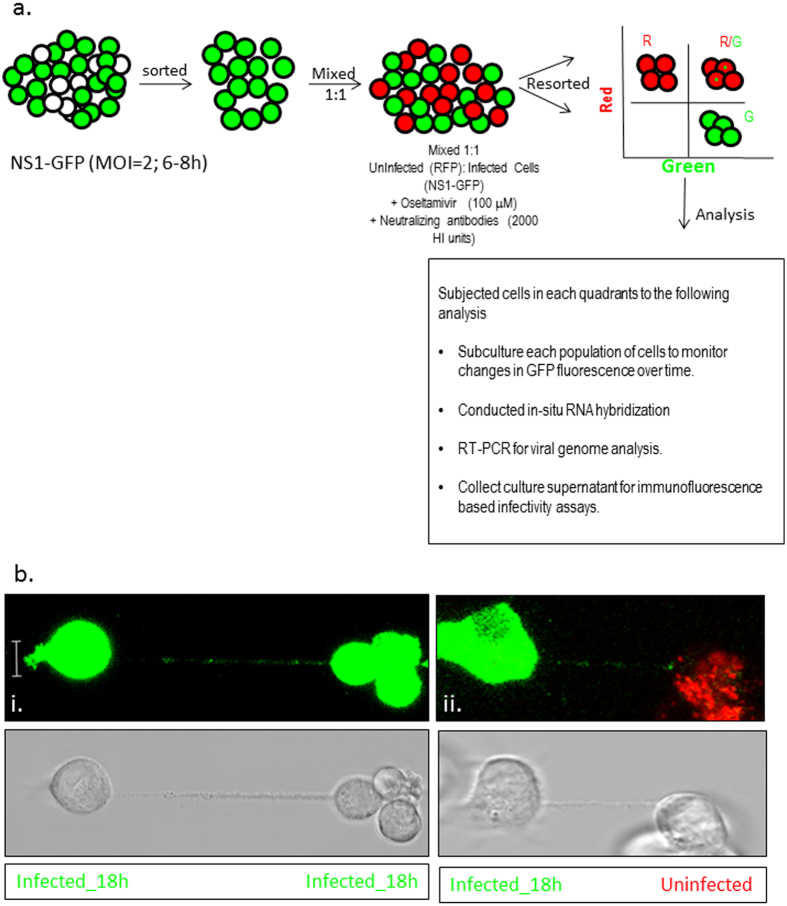
Two population model to evaluate spread of the viral proteins and genome via TNTs. (**a**) Schematics of the two population model design and its application in tracking the fate of the recipient (uninfected) cells. Cells were sorted 6 h after infection with IAV expressing the NS1-GFP construct, mixed with an equal number of RFP-A459 cells, and co-incubated at 37 °C for an additional 4–6 h. The co-incubated cells were then sorted into populations expressing detectable RFP only (R-quadrant), GFP only (G-quadrant), or RFP plus GFP (i.e. initially-uninfected RFP-A549 cells that also expressed NS1-GFP) (R/G quadrant). Cells in these three quadrants were subjected to further analysis as described in the schematic. (**b**) Detection of endogenous NS1-GFP transfer in A549 cells through TNTs. NS1-GFP signal is seen as bright puncta in TNTs of A549 cells infected with NS1-GFP virus or in the TNTs connecting uninfected DiD-labelled-A549 cells (red) and NS1-GFP virus infected cells (green).

**Figure 5:**
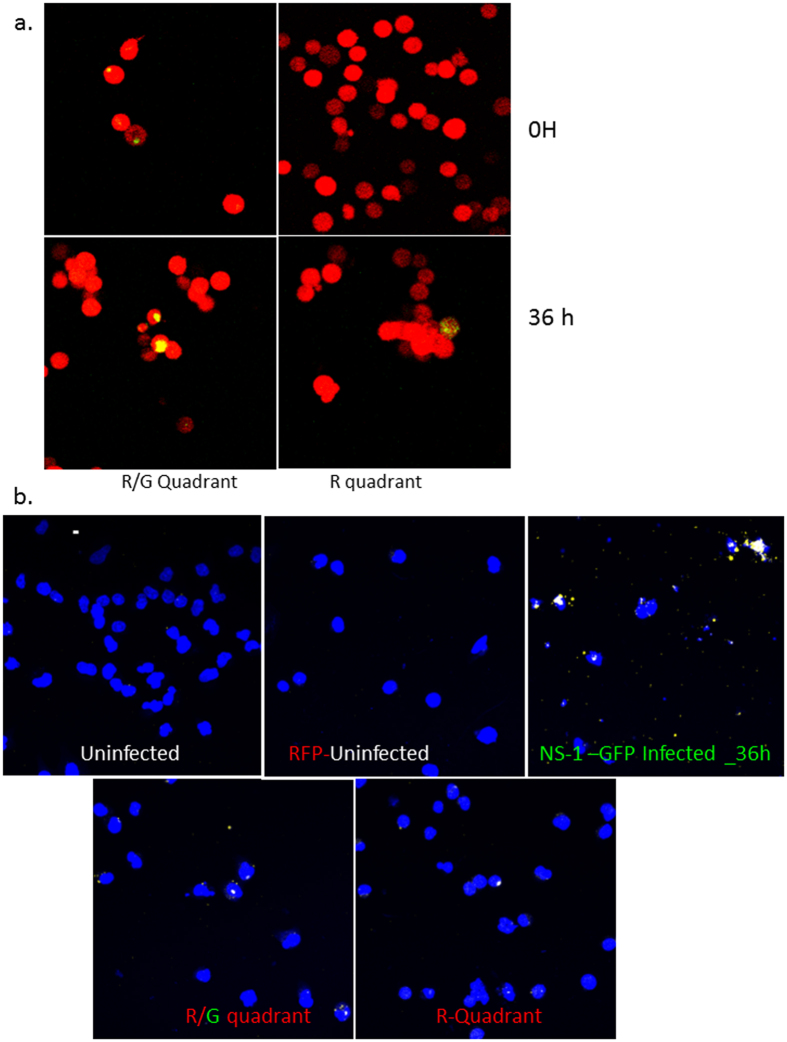
TNTs facilitate transfer of viral proteins and genome. Cells of the R- and R/G- quadrant were cultured for up to 36 h post-sort and the images taken at the indicated time points post-sort. (**a**) Fluorescent images showing expression of NS1-GFP in the cells R/G- or the R-quadrant after incubation at 37 °C for an additional 36 h post-sorting. The cells were also subjected to *in-situ* RNA hybridization. (**b**) *In situ* RNA hybridization in cells of the R/G- or the R- quadrant.

**Figure 6:**
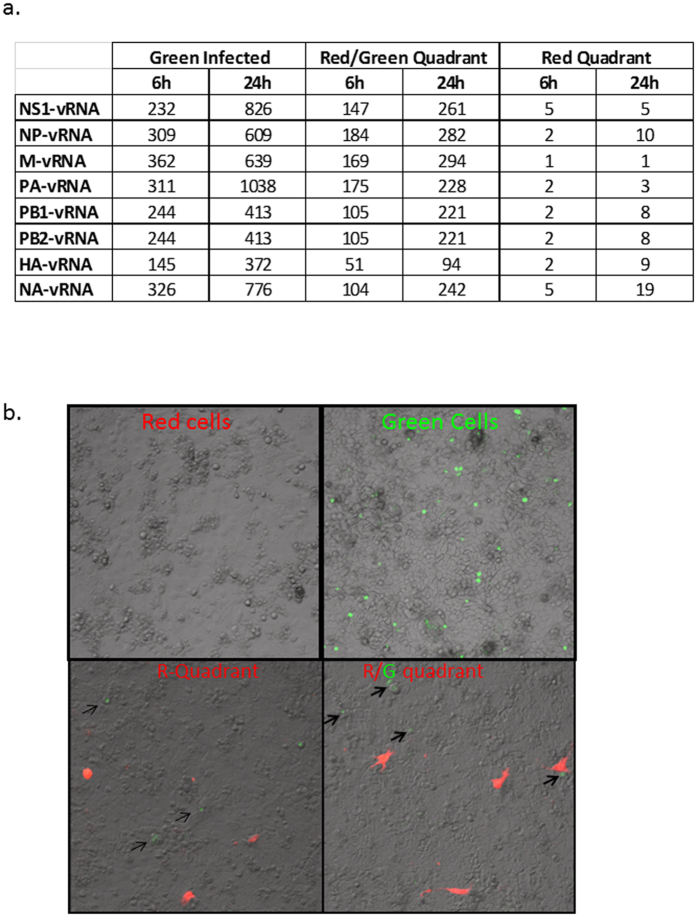
Expression of viral genes in an uninfected population of cells co-cultured with influenza infected cells. Cells of the R- and R/G- quadrant were cultured for up to 36 h post-sort and assays conducted as denoted. (**a**) RT-PCR analysis in cells of the R/G and R quadrant showing fold-increase in expression of viral genes. (**b**) Immunofluorescence-based infectivity assay showing GFP fluorescence in MDCK cells overlaid with cells from the R/G and R quadrants.

Having established that a naïve cell can be connected to an infected cell, we next wanted to examine whether this mechanism of transfer can be used to establish a productive infection in the naïve cells. For this, we set up a two-color population model to differentially track the population of infected cells from the population of uninfected cells (Fig. 4a). We used a recombinant PR8 virus in which Green Fluorescent Protein (GFP) has been fused with the NS1 open reading frame to express NS1-GFP^22^. The use of this virus not only allowed us to track the infected cell population, but also since NS1 is a non-structural protein, ruled out the possibility that the viral proteins observed within the TNTs are from the input virus^23^. We first infected A549 cells with NS1-GFP virus for 1 h and allowed the infection to proceed for 6 h in the presence of the antiviral drug, Oseltamivir, at a concentration of 100 μM. Oseltamivir inhibits the neuraminidase enzyme, which is expressed on the viral surface and is required for the release of the virus from infected cells. Thus in the presence of neuraminidase inhibitors virions stay attached to the membrane of infected cells and are unable to infect neighboring cells^24^. After 6 h of infection, cells expressing the GFP were sorted as described in Materials and Methods. Supplementary Figure 8a shows that the sorted A549-GFP cells are viable for up to 24 h post-sorting. The sorted GFP-expressing cells were mixed with an equal number of naïve Red Fluorescent Protein (RFP) expressing-A549 cells. The RFP-A549 cell line stably expresses the RFP gene and also readily forms TNTs (Supplement Figure 1g). As an additional control to block the spread of the cell-free virus, the uninfected and infected cell populations were co-cultured in the presence of neutralizing antibodies against PR8 virus at 2000 HI units as well as Oseltamivir at a concentration of 100 μM. Under these conditions, cell-free spread of NS1-GFP virus at MOI 0.1–10 was completely blocked (Supplementary Figure 8b). We also serially passaged the supernatants of infected cells in the presence and absence of Oseltamivir and neutralizing antibodies and show that supernatants from cells infected with virus in the presence of Oseltamivir and neutralizing antibodies lacked any infectious particles (Supplementary Figure 8c–e). Having confirmed that naïve cells could only become infected via TNTs in this model, we selectively sorted the naïve cells from the infected cells after 4–6 h of co-culture. As shown in Fig. 4a, we subjected the cells from the different sort quadrants to functional analysis using several different approaches (Fig. 4a). We divided the initially-naive cells (red cells) into two sub-sets during sorting, those that acquired green fluorescence signal within 4 h (referred to as R/G Quadrant) indicating the transfer of influenza virulence factor NS1 protein, and those that had no detectable green signal suggesting the absence of NS1 transfer (referred to as R Quadrant) (Fig. 4a). Cells in the R quadrant may potentially have the viral genome and not the NS1 protein, while the cells in the R/G quadrant would have NS1 protein and the viral genome. TNTs connecting the uninfected and infected cells were observed in the sorted cells suggesting that sorting did not affect the ability of the cells to form TNTs (data not shown). Further, we could clearly recognize NS1-GFP in TNTs of two infected cells (Fig. 4b panel i) or within the TNTs formed between infected (green) and uninfected cells (red) (Fig. 4b, panel ii) suggesting that viral proteins can be easily exchanged via this mechanism. To obtain a robust analysis of infectious viral transfer between cells, we cultured the cells from the R/G or R quadrant for an additional 72 h and monitored changes in these cell populations as compared to naïve cells that were not co-incubated with the infected cells. After sorting, we found that R/G cells had green punctate staining at time 0 h, unlike the R cell population which had no observable green punctate staining at time 0 h post-sort (Fig. 5a). After 36 h of sub-culturing, the frequency of R/G cells expressing punctate green staining increased. Interestingly, at 36 h post-sort, green punctate staining was also observed in cells collected in the R quadrant suggesting this population of cells had received viral contents from the infected cell and that there was active replication of virus in these cells. The percentage of cells with green staining was higher in the R/G quadrant as compared to those in the R-only quadrant (data not shown), and no staining was observed in naïve cells that had not been co-incubated with the sorted green cell population. To demonstrate that the genome can also transfer via this mechanism, we conducted an *in situ* RNA hybridization on the R/G quadrant cells and cells in the R quadrant along with the control cells. As shown in Fig. 5b, we observed NP positive strand RNA in cells in the R/G quadrant, R quadrant, and in the control infected cells (Fig. 5b). The single color of these panels are shown in Supplementary Figure 9a. These results were also confirmed using RT-PCR analysis, where we observed PCR-detectable viral mRNA levels for all the viral genes in the cells of the R/G or the R quadrant 6 h and 24 h after sub-culturing of post-sorted cells (Fig. 6a). At 6 and 24 h post-sorting, the expression of the viral genes was higher in the cells of the R/G quadrant compared to cells in the R quadrant (Fig. 6a). One potential explanation is that cells in the R/G quadrant had acquired the virulence factor NS1-GFP which suppressed the anti-viral innate immune pathway(s) in the cells and thus allowed for productive viral replication. Further, we also observed that expression of the viral genes in the R/G quadrant increased over time (compare expression levels between 6 and 24 h post-sorting). Together, data from the RT-PCR and the RNA *in situ* hybridization experiments suggest that TNTs facilitate viral genome transfer. In parallel, we also cultured the cells of the R/G and the R quadrant in the presence of Oseltamivir and neutralizing antibodies for an additional 6 h and 24 h post-sorting and show active viral replication in the sorted cells via plaque analysis and RT-PCR (Supplementary Figure 9b and c). These results show that the virus exploits TNTs and can replicate within the recipient cells in the presence of neutralizing antibodies and Oseltamivir as seen by the fold increase in levels of viral mRNA at 24 h when compared to expression at 6 h post-sorting (Fig. 6a and Supplementary Figure 9b and c). In accordance with the RT-PCR data, we also collected the supernatants from cells in the R/G quadrant or the R quadrant and infected MDCK cells. We specifically monitored the MDCK cells (white) that were green, as this would indicate infection of the MDCK cells with a live virus. In Fig. 6b, we show green staining in MCDK cells 24 h post-infection with supernatants from the R, or the R/G quadrant. These findings indicate that cells in the R/G quadrant or the R quadrant had active viral replication, and demonstrate that uninfected cells can become infected via transfer of influenza virus genome and proteins from adjacent infected cells even in the absence of extracellular spread of virus.

### TNT formation and transfer of viral genome is attenuated in the presence of inhibitors of actin polymerization

**Figure 7:**
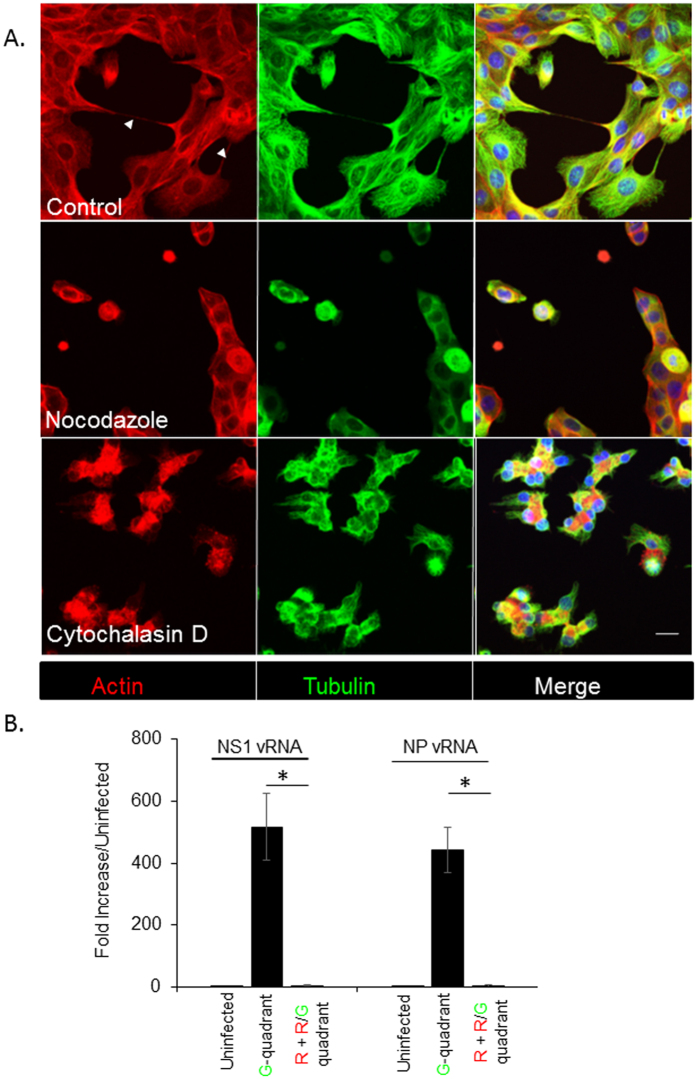
Pharmaceutical drugs that inhibit cytoskeletal dynamics attenuate the number of TNTs and cell-to-cell spread of the viral genome. (**A**) A549 cells were incubated for 6 h with or without cytochalasin D and nocodozole, and stained for actin (Red) and Tubulin (Green) filaments before imaging and recording in 3D by confocal microscopy. Shown are the maximum intensity projections of the focal planes of a representative image stack from control and drug-treated cells. Arrows indicate TNTs observed in control (DMSO-treated) cells. TNTs were not observed in cells treated with either drug compared to the untreated-control. For each time point > 1000 cells were analyzed, and the experiment was repeated three times. Scale bar: 10 μm. (**B**) NS1-GFP infected cells were incubated with uninfected RFP-A549 cells, in the presence of neutralizing antibodies (2000 HI units), Oseltamivir (100 μM) and Cytochalasin D (20 μM). After 8 h of incubation, the cells in the R-and R/G-quadrants and G-quadrant were sorted. The sorted cells were cultured in the presence of Cytochalasin D (20 μM) for an additional 6 h, after which RNA was extracted and the expression of vRNA for NP and NS1 were analyzed using RT-PCR. The graph shows the expression of vRNA genes, NP and NS1 and data are shown as mean ± SD. β-Actin was used as a loading control. ANOVA was performed to compare the expression in cells in the G-quadrant versus cells in the R- and R/G-quadrants, and p values < 0.05 are indicated with an asterisk.

Because F-actin and tubulin proteins were found in TNT structures, we next examined whether the inhibitors of actin and tubulin polymerization would impact TNT formation as well as the transfer of viral proteins and/or genome. Trypsinized cells were seeded in the presence or absence of Cytochalasin D, a well characterized inhibitor or actin polymerization, or Nocodazole which is known to inhibit microtubule assembly, and TNT formation was examined 6 h after seeding using immunofluorescence microscopy. Analysis of optical sections of the cells reveal that formation of TNTs was attenuated in the presence of the drugs (Fig. 7A). To address whether disruption of actin polymerization also affects the TNT-mediated transfer of influenza viral proteins and genome, we used a similar format of the experiment as described in Fig. 4a. A population of uninfected RFP-A549 cells was co-incubated with A549 cells infected with the NS1-GFP virus in the presence of 2000 HI units of neutralizing antibodies, 100 μM of Oseltamivir, and 20 μM Cytochalasin D. After 8 h of co-incubation, cells in the R-quadrant and R/G quadrant were sorted and RT-PCR assay was used to assess the presence of viral genome. Unlike our previous results that demonstrated viral genome and protein transfer in the presence of neutralizing antibodies and anti-viral drug Oseltamivir (Fig. 6a), in the presence of Cytochalasin D, neither NS1 nor NP vRNA was detected in the cells of the R and R/G quadrant (Fig. 7B). Overall, these data indicate that TNT formation requires actin and the microtubular network, and the transfer of the viral genome in the presence of neutralizing antibodies requires actin-based TNT structures. These results with Cytochalasin D are consistent with the data published by Roberts *et al*., who demonstrated the transfer of viral proteins in the presence of antiviral drug, Zanamivir (in the case of influenza) and virus neutralizing antibodies (in the case of PIV5) and suggest a role for cytoskeletal networks in the formation of TNT and TNT-mediated transfer of viral genome/proteins from infected to naïve cells^9^. Overall, our results suggest an important role for TNTs in spread of influenza virus.

Influenza virus enters the body via infection of epithelial cells and it is important to understand how they spread from the infected cells to adjoining cells. By tracking the fate of uninfected cells that are incubated with infected cells, we reveal a novel mechanism of cell-to-cell transmission of influenza virus, which potentially involves the transport of viral proteins as well as viral genome through the TNTs resulting in viral replication in the recipient cell. Hijacking an existing intercellular network for spreading within tissue offers several advantages to the virus. Firstly, the influenza virus could potentially overcome host anti-viral innate immune defenses in the neighboring cells by the TNT-mediated transfer of virulence factor, NS1, which is known to attenuate the antiviral state of the cell and inhibit pathogen sensors like retinoic acid inducible gene-I^25^,^26^,^27^,^28^,^29^. As shown here, we observed differential viral gene accumulation in the cells that had received NS1 protein (cells in R/G quadrant) compared to cells that had no detectable levels of NS1 protein (cells in R quadrants). Additionally, the virus can evade recognition by pre-existing antibodies, which could otherwise neutralize the newly released virus from the infected cells and prevent it from infecting neighboring cells. The mechanistic insight obtained from this work will contribute to the development of novel anti-viral strategies.

## Materials and Methods

### Cell culture and treatments

Madin-Darby canine kidney (MDCK-London) and Human Alveolar Lung Epithelial cells (A549) were maintained in Dulbecco’s modified Eagle’s medium (DMEM) supplemented with 10% fetal bovine serum (FBS), 2 mM L-glutamine (Life Technologies) and 100 U/ml Penicillin-Streptomycin (P/S) (Life Technologies). All cell lines were maintained in a humidified incubator containing 5% CO_2_ at 37 °C and sub-cultured when the cells attained 70–80% confluency. A549 cell lines stably expressing Red Fluorescent Protein (RFP) or the Green Fluorescent Protein (GFP) were obtained from AntiCancer Incorporated and Cell BioLabs, respectively, and cultured in complete DMEM media containing 10% FBS, 0.1 mM MEM Non- Essential Amino Acids, 2 mM L-glutamine, and 100 U/ml P/S (Life Technologies). Cells were infected with the NS1-GFP virus as described^22^. A549 cells were treated with Cytochalasin D or Nocodazole at a concentration of 20 μM or 30 μM as previously described^9^.

### Viruses

Influenza viruses used in this study include A/Puerto Rico/8/34 [(PR8); H1N1] obtained from the Influenza Reagent Resource (Manassas, VA) and NS1-GFP virus was provided by Adolfo Garcia-Sastre, Mount Sinai School of Medicine, New York City, NY. Viruses were propagated for 2 d in the allantoic cavity of 10 d old embrionated chicken eggs. Pooled allantoic fluid was clarified by centrifugation, aliquoted, titered and stored at −80 °C until use.

### Reagents

1, 1′-dioctadecyl-3, 3, 3′, 3′-tetramethylindodicarbocyanine perchlorate (Did) and 3, 3′-dioctadecyloxacarbocyanine perchlorate (Diol) were obtained from Life Technologies. Oseltamivir carboxylate was purchased from Sequoia Research Products Ltd. Cytochalasin D and Nocodazole were purchased from Sigma Aldrich.

### Antibodies and Generation of influenza-immune sera

Antibodies specific for influenza virus included mouse monoclonal anti-influenza A virus NP antibody, obtained from the Influenza Reagent Resource; anti-HA (FR-572, World Health Organization Mouse Monoclonal Antibody Influenza Type A (H1)); anti-M2 (Sc-32238, Santa Cruz), anti-M (M2-1C6-4R3 (ATCC® HB-64™)), anti-tubulin (Sc32293, Santa Cruz), anti-tubulin (Cat#7634, Cell Signaling), Goat anti-Rabbit or anti-mouse IgG (H + L) Secondary Antibody, Alexa Fluor® 488 or 549 conjugate (Thermo Scientific).

Female BALB/c mice, 6–8 wk old, were purchased from The Jackson Laboratory (Bar Harbor, ME). Ten 3-month-old BALB/c mice were bled to provide pre-immune serum and were infected via the intraperitoneal route of infection with 400 HA units of PR8 virus in 0.2 ml of PBS. Mice were inoculated twice at an interval of 4–5 wk. After the second inoculation, sera were collected every 2 wk and analyzed for hemagglutination inhibition (HAI) titer. Serial dilutions of Receptor Destroying Enzyme (RDE-II) treated sera were mixed with 8 HA/50 μl of egg grown NS1-GFP virus. Mixtures of serum dilutions were incubated for 15 min, followed by addition of 50 μl of 0.5% Turkey red blood cells (Innovative Research). The highest serum dilution inhibiting hemagglutination was taken as the HI titer. All animal research was performed in accordance with the recommendations in the Guide for the Care and Use of Laboratory Animals of the National Institutes of Health. Mice were housed in a specific pathogen-free environment in an Association for Assessment and Accreditation of Laboratory Animal Care International-accredited facility at the Centers for Disease Control and Prevention under the guidance of the Centers for Disease Control and Prevention’s Institutional Animal Care and Use Committee (IACUC, animal welfare assurance number A4365-01). Animal research was approved by the CDC–IACUC and was conducted in an Assessment and Accreditation of Laboratory Animal Care International-accredited facility.

### Virus infection

Infection of MDCK cells or A549 cells with influenza viruses was performed as previously described^30^. The cells were incubated with viruses diluted in DMEM supplemented with 1% bovine serum albumin (BSA) and 1% P/S for 60 min. After virus adsorption, the cells were washed and the post-infection media (DMEM supplemented with 0.3% BSA, 1% P/S, and 1 μg/ml TPCK (L-1-tosylamide-2-phenylethyl chloromethyl ketone)-trypsin) pre-warmed to 37 °C was added immediately to the cells. The cells were transferred to 37 °C to allow virus entry. For cells treated with Oseltamivir during infection, the compound was present in both the infection media and post-infection media at a concentration of 100 μM Oseltamivir (Sequoia).

### Cell membrane labeling and live cell imaging

A549 cells were incubated with 2.5 μM of either DiD or DiO dye per manufacturer’s instructions. Live cells, either stained with DiD or DiO or infected with NS1-GFP virus were imaged in a 35-mm glass bottom dish (MatTek). Culture dishes were mounted on a Zeiss LSM710 Confocal microscope equipped with a heated live-cell incubation chamber maintained at 37 °C and 5% CO_2_ and were imaged using excitation wavelengths of 488, 568, or 633 and a 100 × oil objective. Image analysis was performed using ZenSoftware (Zeiss) or Adobe photoshop (Adobe). Brightness and contrasts were changed in some images only to increase visibility of nanotubes.

### Cell culture preparation for expansion microscopy

Expansion microscopy was performed as described^13^ with modification. A549 cells were seeded on a glass bottom plate, 4 h after seeding, cells were fixed in 3% formaldehyde/0.1% glutaraldehyde for 10 minutes followed by quenching in 100 mM glycine for 10 minutes. Cells were infused with monomer solution (1× PBS, 2 M NaCl, 8.625% (w/w) sodium acrylate, 2.5% (w/w) acrylamide, 0.15% (w/w) N,N’-methylenebisacrylamide). Concentrated stocks (10% w/w) of ammonium persulfate (APS) initiator and tetramethylethylenediamine (TEMED) accelerator were added to the monomer solution up to 0.2% (w/w) each. Cells were incubated with at least 100-fold excess volume of monomer solution and transferred to a humidified 37 °C incubator for 1 h. Infused samples were placed in excess volumes of double distilled water and allowed to expand for 1–1.5 h. After expansion cells were imaged using the LSM710 microscope (Zeiss).

### Immunofluorescence Microscopy

A549 cells were infected with PR8 virus or the NS1-GFP PR8 virus at a MOI of 1 for the indicated times as shown in results. Infection was synchronized by allowing virus to attach at 37 °C for 1 h followed by washing cells in phosphate-buffered saline (PBS) and overlaying the monolayer with complete DMEM media. The following day infected cells were trypsinized and seeded on glass-bottom dishes for 6 h. To stain influenza virus proteins, cells were fixed with 10% formaldehyde, permeabilized with PBS plus 0.1% Tween-20, and stained with the primary antibodies at a concentration of 1:1000. The secondary antibodies were goat-anti mouse or anti-rabbit conjugated Alex Flour 594 or 488 at a concentration of 1:1000. After room temperature incubation for 1 h with secondary antibodies, coverslips were washed in PBST, and mounted onto glass slides using Prolong Gold (Invitrogen) and left to set overnight in the dark. Stained cells were imaged with SlowFade Gold (Invitrogen) antifade reagent using a Zeiss Laser Scanning Confocal microscope (LSM710) with 100×1.30 NA oil objective at 1 Airy unit and Nyquist sampling.

### Transmission Electron Microscopy

Rectangular glass cover slip were cut into small pieces and dropped in a 6-well culture dish. MDCK cells were seeded in the 6-well culture dish for 6 h. Cells were washed in phosphate buffer (pH7.3) and fixed in buffered 2.5% glutaraldehyde. After 1 h, the glutaraldehyde was removed and replaced with phosphate buffer. Cells were not scraped from the cover slips but were processed in the 6-well culture dish. The cells were post fixed in 1% osmium tetroxide, rinsed with water and en-bloc stained with 4% uranyl acetate (UA). After rinsing the UA, the cells were dehydrated in a graded ethanol series. After five exchanges of 100% ethanol, cells were infiltrated with resin using 1:2, 1:1, and 2:1 ratios of 100% ethanol to Epon-substitute/Araldite mixture^31^. After four exchanges of 100% resin, cells were polymerized at 60 °C overnight. Resin-embedded cells were evaluated under a compound microscope to identify areas containing nanotubes. Selected areas were trimmed from the surrounding resin-embedded material and mounted on blank blocks. Ultrathin sections were cut and stained with 4% UA and lead citrate prior to reviewing by transmission electron microscope (Tecnai BioTwin, FEI Company, Hillsboro, OR). Cells connected with TNTs were observed and imaged using a 2 K by 2 K camera (AMT, Danvers, MA).

### Scanning Electron Microscopy

For scanning electron microscopy (SEM), square glass cover slips (Zeiss) were cut into small sections and MDCK cells seeded on them for 6 h. After culturing, samples were washed twice for 5 min each with fixation buffer (2.5% glutaraldehyde in 0.1 M cacodylate buffer). Samples were post-fixed in 1% osmium tetroxide in 0.1 M cacodylate buffer for 1 h. Samples were washed with two 5 min changes of de-ionized water then dehydrated through an ethanol series to three changes of dry 100% ethanol using 5 min per step. Samples were transferred to labeled capsules in the carrier boat for the critical point drying unit (Polaron E3100) filled with dry 100% ethanol. The carrier boat was placed into the CPD unit and sealed and the chamber filled with liquid CO_2_. The ethanol was gently exchanged for liquid CO_2_ by a continuous flow of liquid CO_2_. When the ethanol was completely exchanged for CO_2_, the CO_2_ was brought to its critical point of 31 °C and 1072 psi then allowed to slowly evaporate. The dried samples were removed from the CPD unit, secured to labeled SEM stubs and sputter coated with chromium at a thickness of 10 nm using a Denton DV-602 magnetron sputter coater. The coated samples were imaged in-lens in the upper stage of a Topcon DS150 field emission SEM at 5 kV and 10 kV.

### Fluorescent labelling and tracking of mitochondria

A549 cells were labelled with 5 μM mitotracker green or mitotracker red (Life Technologies), leading to differentially fluorescently labelled mitochondria in each population. For measuring the exchange of mitochondria between the two populations, differentially labeled cells were mixed 1:1 and co-cultured for 6 h. After 1 h, when the cells were attached to the culture dish, the medium was exchanged with fresh medium. Cells were inspected with Differential interference contrast (DIC)- and fluorescence microscopy. Cells connected by TNTs were examined for mitochondrial content.

### Fluorescence-activated cell sorting (FACS)

A population of A549 cells forming an adherent non-confluent monolayer was incubated with NS1-GFP virus at a MOI of 5.0 for 1 h in infection media. After 1 h of infection with virus, the cells were washed once with DMEM supplemented with 0.1% BSA and cultured for an additional 4–6 hr at 37 °C with 5% CO_2_ in complete DMEM media containing 10% FBS, 10 U/ml of P/S to which 100 μM Oseltamivir was added. Cells were trypsinized by adding (0.5%) trypsin at 37 °C for 10 minutes followed by pelleting and resuspension in DMEM culture medium with 5% FBS. The cell suspension was passed through a 70-μm cell strainer and cells subjected to sorting using a LSRII machine (BD) equipped with a 43 air-cooled 488 nm argon laser at 100 mW of power. The Diva program (BD) was used to set up the experimental parameters. Cells were > 99% pure after sorting (Supplementary Fig. 3a). Post-sorting, the GFP-expressing cells were collected in FBS, washed with complete DMEM media, mixed 1:1 with RFP-A549 cells, and plated in 6-well plates (Corning) at a concentration of 100,000–150,000 cells/well. Incubation media consisted of complete DMEM supplemented with 10% FBS, 10U/Ml P/S, 5 mM L-Glutamine, 100 μM Oseltamivir, and 1:50 dilution of the of neutralization sera. After 4–6 h of co-incubation, cells were trypsinized, washed twice with DMEM, and prepared for sorting as described above. Cells were collected as shown in Fig. 3B. Cells in the different quadrants were cultured separately and used for various assays as described in Fig. 3B. The two-color populations of cells were photographed at 100x magnification using the LSM710 confocal microscope.

### Immunofluorescence RNA

These experiments employ the QuantiGene ViewRNA assay kit from Panomics and samples were processed as previously described^32^.

### Immunofluorescence based infectivity assay

Productive infectious particles from the sorted population of cells was assessed by using a modification of the plaque assay method as described^33^. Sorted cells seeded in a 24-well plate were overlaid with 5 × 10^4^ MDCK cells. The plates were incubated for 24–48 h and accumulation of GFP in MDCK cells was monitored following image capture.

### RNA extraction, RT-PCR and real-time PCR

Total RNA were extracted using Trizol reagent (Invitrogen,Carlsbad, CA) according to the manufacturer’s instructions. 2 μg of total RNA were used for cDNA synthesis using Superscript II Reverse Transcriptase (Life Technologies) according to the manufacturer’s directions. Real time RT-PCR was conducted using a Stratagene Q3005 PCR machine for mRNA expression of NS1 and β-actin. Parallel reactions without reverse transcriptase were included as negative controls. Reverse transcription reactions (1/50th of each reaction) were analyzed using SYBR green Q-PCR reagents (Stratagene). PCR condition was kept as 94 °C for 15 s, annealing at 56 °C for 30 s, and extension at 72 °C for 30 s for a total of 45 cycles. The threshold cycle number for cDNA was normalized to that of β-actin mRNA, and the resulting value was converted to a linear scale. The experiment was repeated twice and results from one experiment shown. The primer sequences for NS1, NP, M, PA, HA, NA, PB1 and PB2 are described and published^34^,^35^,^36^.

### Quantification of TNTs

For quantification of TNTs upon infection, A549 cells were infected with PR8 virus at an MOI = 1.0 for 18 h, trypsinized and plated for an additional 2–4 h at a density of 500,000cells/well in a 6-well plate. Cells (500) were counted and imaged for TNT connection using the confocal LSM 710 Microscope. To investigate the effect of Nocodazole and Cytochalasin D on TNT formation, A549 cells were plated in the presence or absence of drug for 6 h and cells were fixed and stained with Alexa594-Phalloidin for staining actin. Cells were also co-stained for tubulin filaments using anti-tubulin antibody (Sc32293, Santa Cruz) (1:500) followed by anti-mouse antibodies conjugated to Alexa Fluor 488 (green), and nucleus was stained with DAPI (blue). Cells were mounted and a total of 500 cells for each condition were counted for TNT number under control and drug-treated conditions.

### Statistical Analysis

Students t-tests as well as ANOVA were used as statistical tests to compare two or more than two groups respectively and error bars are shown as ± SD.

## Additional Information

**How to cite this article**: Kumar, A. *et al*. Influenza virus exploits tunneling nanotubes for cell-to-cell spread. *Sci. Rep.* **7**, 40360; doi: 10.1038/srep40360 (2017).

**Publisher's note:** Springer Nature remains neutral with regard to jurisdictional claims in published maps and institutional affiliations.

## Supplementary Material

Supplementary Movie 1

Supplementary Movie 2

Supplementary Movie 3

Supplementary Movie 4

Supplementary File
