# Supplementary material for: Influenza virus exploits tunneling nanotubes for cell-to-cell spread
Source: Sci Rep. 2017 Jan 6;7:40360. doi: 10.1038/srep40360 (PMC5216422; doi:10.1038/srep40360)
Supplement: Supplementary File [file srep40360-s5.pdf]

Influenza virus exploits tunneling nanotubes for cell-to-cell spread

Amrita Kumar[1], Jin Hyang Kim[1], Priya Ranjan[1], Maureen G. Metcalfe[2], Weiping Cao[1], Margarita Mishina[1], Shivaprakash Gangappa[1], Zhu Guo[3], Edward S. Boyden[4], Sherif Zaki[2], Ian York[1], Adolfo García-Sastre[5], Michael Shaw[6] and Suryaprakash Sambhara[1*]

[1]Immunology and Pathogenesis Branch, Influenza Division, Centers for Disease Control and Prevention, 1600 Clifton Road, Atlanta, GA 30329-4027, USA.

[2]Infectious Diseases Pathology Branch, Division of High-Consequence Pathogens and Pathology, Centers for Disease Control and Prevention, 1600 Clifton Road, Atlanta, GA 30329-4027, USA.

[3]Virus Surveillance and Diagnostics Branch, Influenza Division, Centers for Disease Control and Prevention, 1600 Clifton Road, Atlanta, GA 30329-4027, USA.

[4] Media Lab, McGovern Institute, Department of Brain and Cognitive Sciences, MIT, Cambridge, MA, USA.

[5]Department of Microbiology, Department of Infectious Disease, Global Health and Emerging Pathogens Institute and Department of Medicine, Division of Infectious Diseases, Icahn School of Medicine at Mount Sinai, New York.

[6]Office of Infectious Diseases, Centers for Disease Control and Prevention, 1600 Clifton Road, Atlanta, GA 30329-4027, USA.

[*]Address correspondence to ssambhara@cdc.gov

Disclaimer

The findings and conclusions in this report are those of the authors and do not necessarily represent the views of Centers for Disease Control and Prevention.

SUPPLEMENTAL FIGURE LEGENDS

Supplementary Figure 1. TNTs connect neighboring A549 cells. (a and b) 3D-live microscopy image showing TNT connecting neighboring A549 cells in a non-confluent monolayer (a) and a confluent monolayer (b). For both panels a and b, A549 cells were seeded on a glass-bottom culture dish for 4-6 h and images captured with a Zeiss confocal microscope equipped with a 100X /1.4 NA oil objective. (c) Average length of the TNTs in A549 cells. TNTs (n>250) were analyzed from more than three independent experiments. Percent of cells are plotted as function of the length of the TNTs, showing the heterogeneous nature of TNTs. (d and e) A549 cells connected by a curved (d) or branched (e) TNT, suggesting TNTs can grow in a 3D environment. Cells in (d) were stained with Alexa-594 phalloidin after fixation. (f and g) A549 cells lines stably transfected with either GFP (f) or RFP (g). Cells in e, f, and g were grown on glass bottom culture dish and imaged under live conditions.

Supplementary Figure 2. Characterization of TNTs in MDCK and A549 cells. (a) Three-dimensional analysis of TNT in A594 labelled with Alexa405-phallodin and as analyzed by depth analysis (Zeiss software). Color represents z-depth "standing up" on coverslip or the cell along the z-axis. Red indicates the glass cover slip; and blue, indicates distance furthest from the glass bottom. (**b**) TNT connecting two neighboring MDCK cells labelled with DiD and imaged under live conditions. (**c**) TNT connecting two neighboring A549 cells labelled with either the DiD (red) or DiO (green) dyes and co-incubated together before imaging.

Figure 3. **Ultra-structure of TNTs in MDCK cells.** (**a**) Scanning electron microscopy (SEM) shows the ultrastructure of a TNT between two MDCK cells. Scale bar corresponds to 2 μm. (**b**) A TEM section of MDCK cells cultured for 4 h after seeding, indicates the presence of TNTs

connecting neighboring MDCK cells. The inset within shows lower magnification image of the TNT connecting two neighboring cells.

Supplementary Figure 4. TEM images show bundles of fibers running along the length of the TNT. Higher magnification panels of the TNT as observed in MDCK cells by TEM. Blue arrows show bundles of fibers running along the length of the TNT.

Supplementary Figure 5. Characterization of TNTs in A549 and MDCK cells. (a) TNTs contain actin and tubulin. Fixed sample of A549 cells stained with phalloidin (green) and anti-tubulin antibody (red). (b) A549 cells stained with DiD (red) mixed with unstained cells, was imaged for a period of 6 h under live conditions. Panels shows TNTs at 0 h (start time of imaging) and 4.5 h (time post imaging). TNTs shows a continuous connection between the two cells with punctate red staining in unstained cells. (c) SEM images of TNT before (left) and after (right) exposure to a high resolution scan. A breakage in the TNT was observed as it was being scanned.

Supplementary Figure 6. TNT like structure showing presence of mitochondria and ribosomes. TEM image showing mitochondria and ribosome within a TNT-like structure. In this TEM grid, the cell was captured at one end of the TNT. Panels i-iv show higher magnifications images of the TNT shown in inset in panel i.

Supplementary Figure 7. Tunneling nanotubes serve as a conduit for intracellular transport of IAV proteins. Fluorescent image of TNTs, shown in Fig 3, with color representing z-depth “standing up” on coverslip. The color coding denotes the cell along the z-axis. Red indicates the

glass cover slip; and blue, indicates distance furthest from the glass bottom. The left panel for each viral protein shows the top stack and the right panel shows the bottom stack along the z-axis.

Supplementary Figure 8. Two population model to evaluate spread of the viral proteins and genome via TNTs. (**a**) Panel showing a population of sorted NS1-GFP A549 cells. A549 cells infected with NS1-PR8 were sorted 6 h post-infection and cultured in complete media and monitored for upto 24h to ensure that cells were viable and free of contamination. (**b**) Panel showing infectivity of the NS1-GFP PR8 virus in the presence of 2000 HI units of neutralizing antibodies and Oseltamivir (100 μM) as compared to infection in the presence of serum obtained from uninfected mice used in control panels. (**c**) Images showing infectivity of culture supernatants after serial passaging the NS1-GFP virus in A549 cells. (**d**) Plaque assay of culture supernatants from third passage of the NS1-GFP virus. (e) RT-PCR of culture supernatant from third passage of the NS1-GFP virus. For c-e, culture supernatants from A549 infected at an MOI of 1.0 for 96 h, was collected and serially passaged. At the end of the third passage (denoted as P_3), culture supernatants were analyzed for the presence of virus as described denoted. No plaques or message for NP were observed in the presence of Oseltamivir and Neutralizing Antibodies.

Supplementary Figure 9. (a) Detection of viral RNA in A549 cells using RNA fluorescence *in situ* hybridization. After experimental conditions, cells were fixed and processed with the ViewRNA ISH Cell Assay kit and a type6-probe set against nucleoprotein (NP) viral genomic segment (yellow). Blue, denotes DNA stained with DAPI; Red, denotes RFP-A549; green denotes A549 cells infected with the NS1-GFP virus and yellow shows the presence of the viral genomic segment. Plaque assay (b) and RT-PCR (c) in NS1-GFP infected cells grown in the presence of neutralizing antibodies (2000 HI units) and Oseltamivir (100 μM). Uninfected A549-RFP cells

were co-incubated with a NS1-GFP infected cells for 4 h. After co-incubation, the A549-RFP cells were sorted from the green NS1-GFP cells, and cultured in complete media containing Oseltamivir (100 μM) and serum antibodies (2000 HI units) for the indicated times. The culture supernatants from samples were collected and subjected to plaque assay (a), and the remaining cells analyzed for expression of NP mRNA by RT-PCR (b).

Supplementary Movie Legends

Supplementary Movie 1. Projection movie of phalloidin-labelled TNT in A549 cells. Images were obtained using a Zeiss Confocal Microscope and the image processed using depth analysis tool in Zen 2010. Still from this movie is shown in Supplementary Figure 2a. Shown is the entire depth along the Z-axis, starting at the bottom of the substratum (color coded red) and ending with the top of the cell (color coded blue). In this image the stack has a depth of 8 μm and the TNT is colored green demonstrating the TNT is sitting at a depth of 3-5 μm. This demonstrates the connection is not attached to the substratum. The video is played at a speed of 2 frames/sec.

Supplementary Movie 2. Time-lapse microscopy showing that TNTs formation is dynamic with new connections forming over time. A549 cells were incubated in the microscope chamber with temperature and CO_2 control and imaged over time, every 30 mins for 7 hrs. For each time points, multiple Z-stacks were obtained. The movie shows the compressed Z-stack played over time. At time 4 h and 4.5 h new TNTs appear keeping the cell in contact with each other (also shown as static images in supplementary figure 2c). The video is played at a speed of 2 frames/sec.

Supplementary Movie 3. Time-lapse microscopy movie showing that TNTs form between two cells when two cells move apart. A549 cells were incubated in the microscope chamber with temperature and CO_2 control and imaged over time, every 10 mins for 8 h. For each time points, multiple Z-stacks were obtained and the movie is edited in Z-stack and time. Movie shows the formation of TNT between two cells that move apart over time. The video is played at a speed of 2 frames/sec.

Supplementary Movie 4. Time-lapse microscopy showing that TNTs connection are fragile and break under exposure to high laser power. A549 cells stained with DiD (red) were co-incubated and mounted in the microscope chamber with temperature and CO_2 control. Z-stacks was collected every 30 mins for 7 h. Movie was made by compressing the Z-stacks at each time point and edited in time. Stills from this movie are shown in figure 1e.

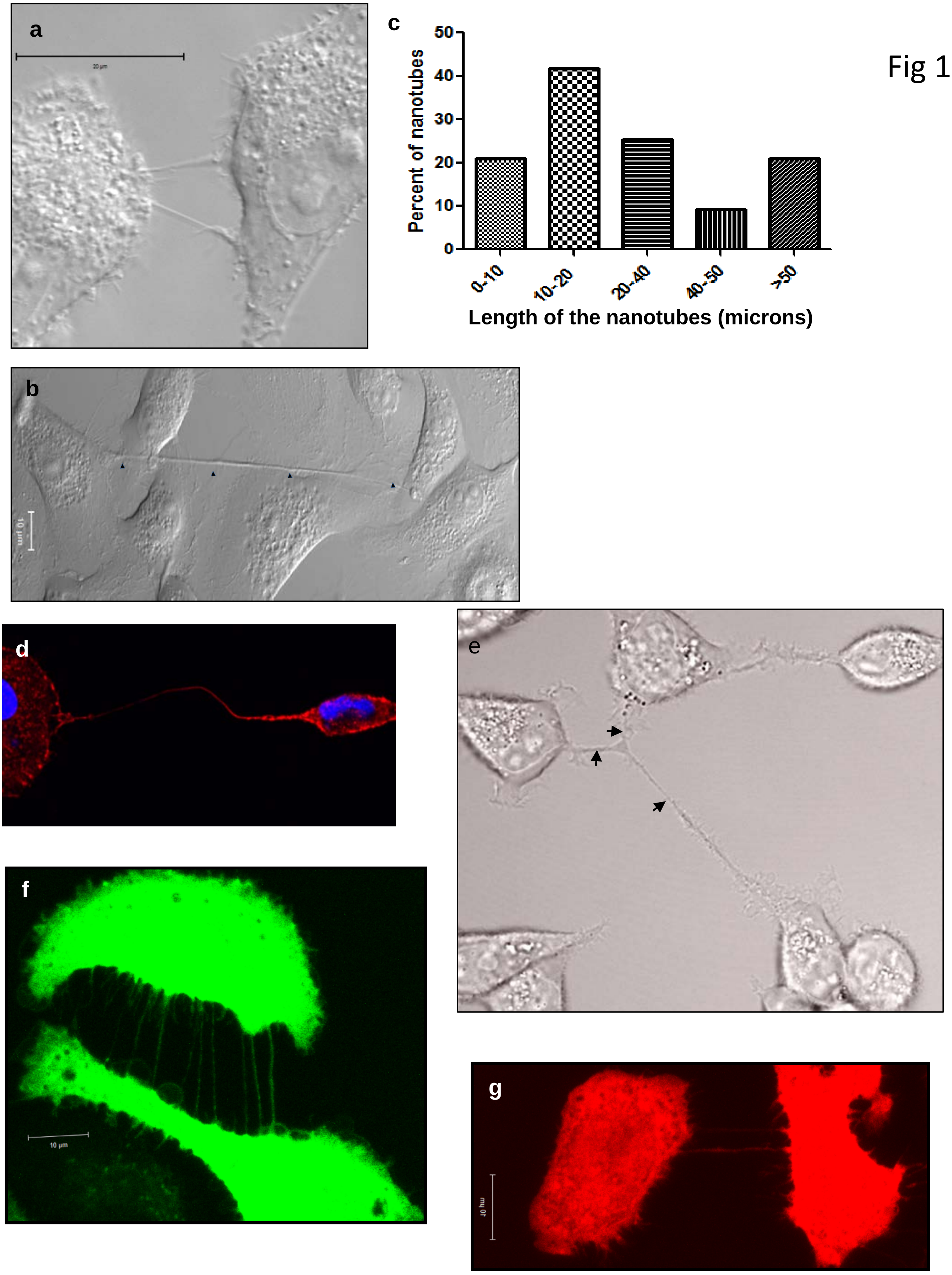

a
20 µm
c
Percent of nanotubes
50
40
30
20
10
0
0-10
10-20
20-40
40-50
>50
Length of the nanotubes (microns)
b
10 µm
d
e
f
10 µm
g
10 µm

Fig 1.

Fig 2.

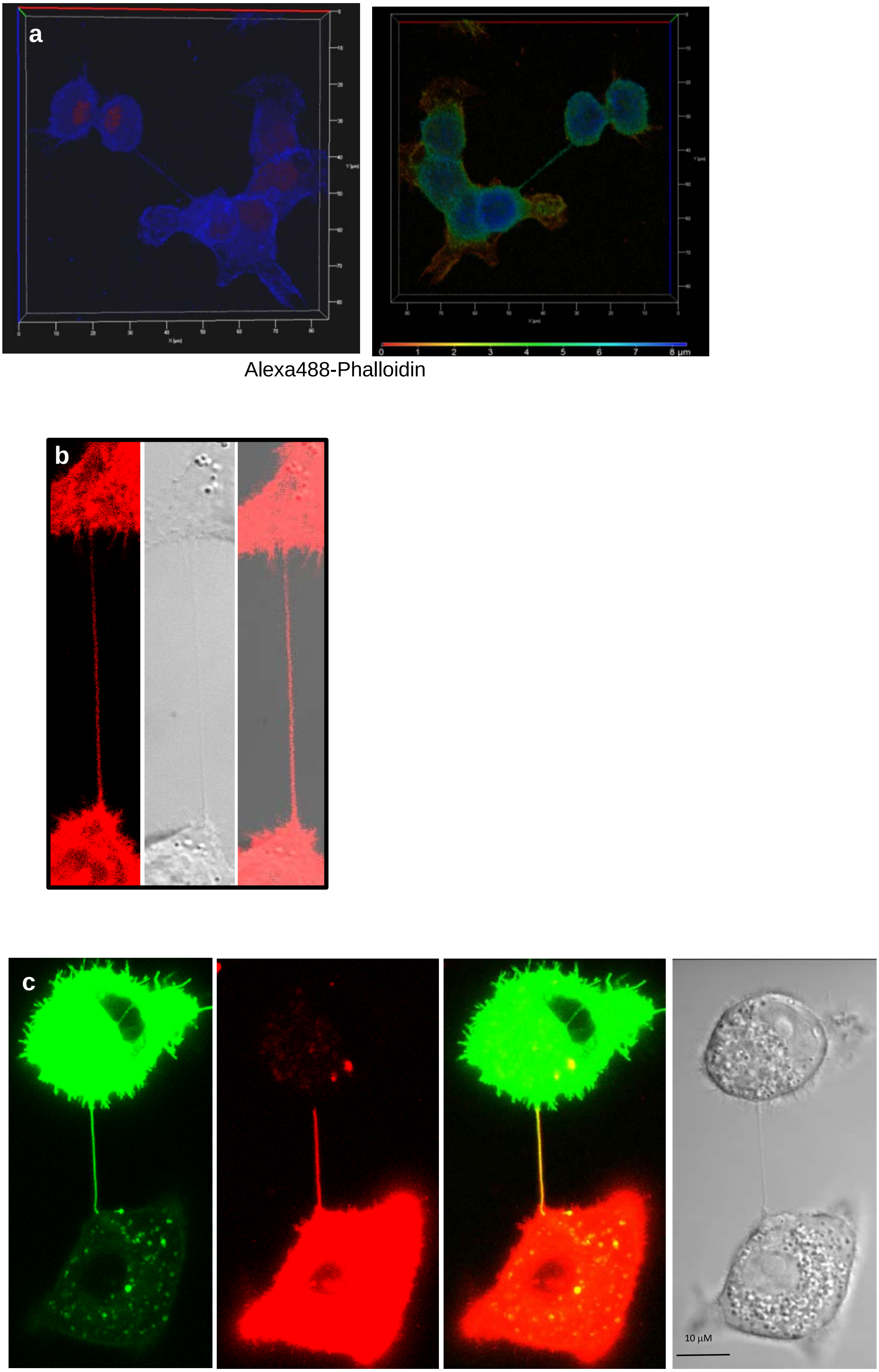

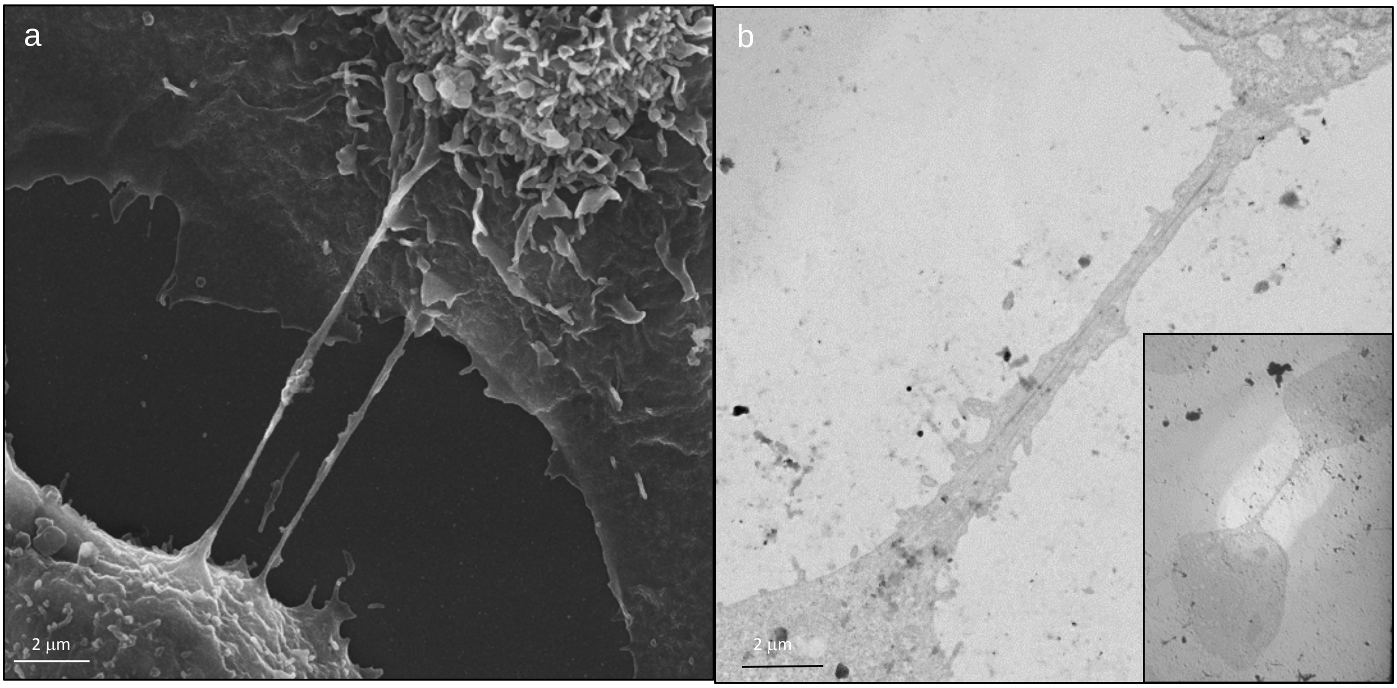

a
2 μm
b
2 μm

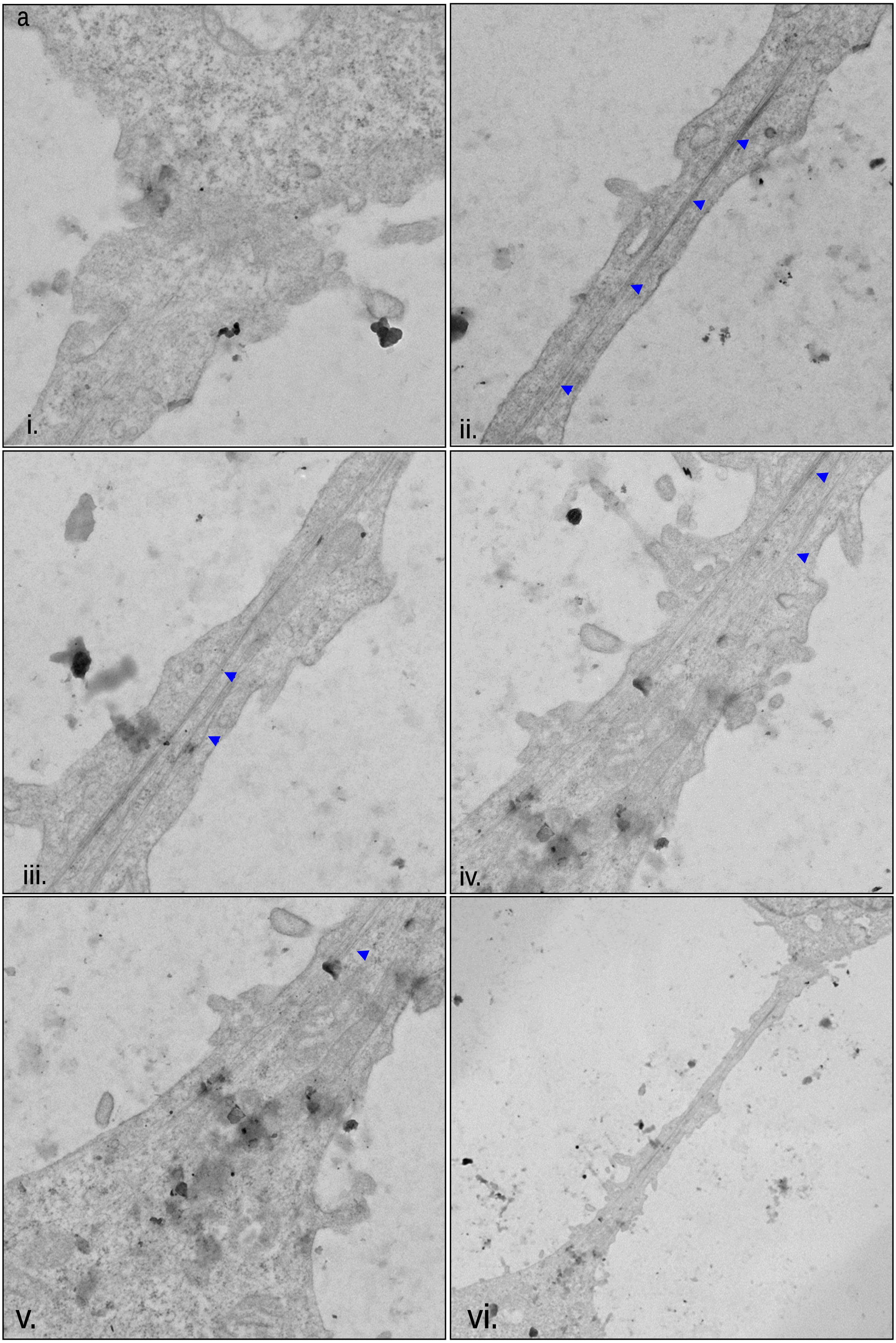

Fig 4.

Fig 5.

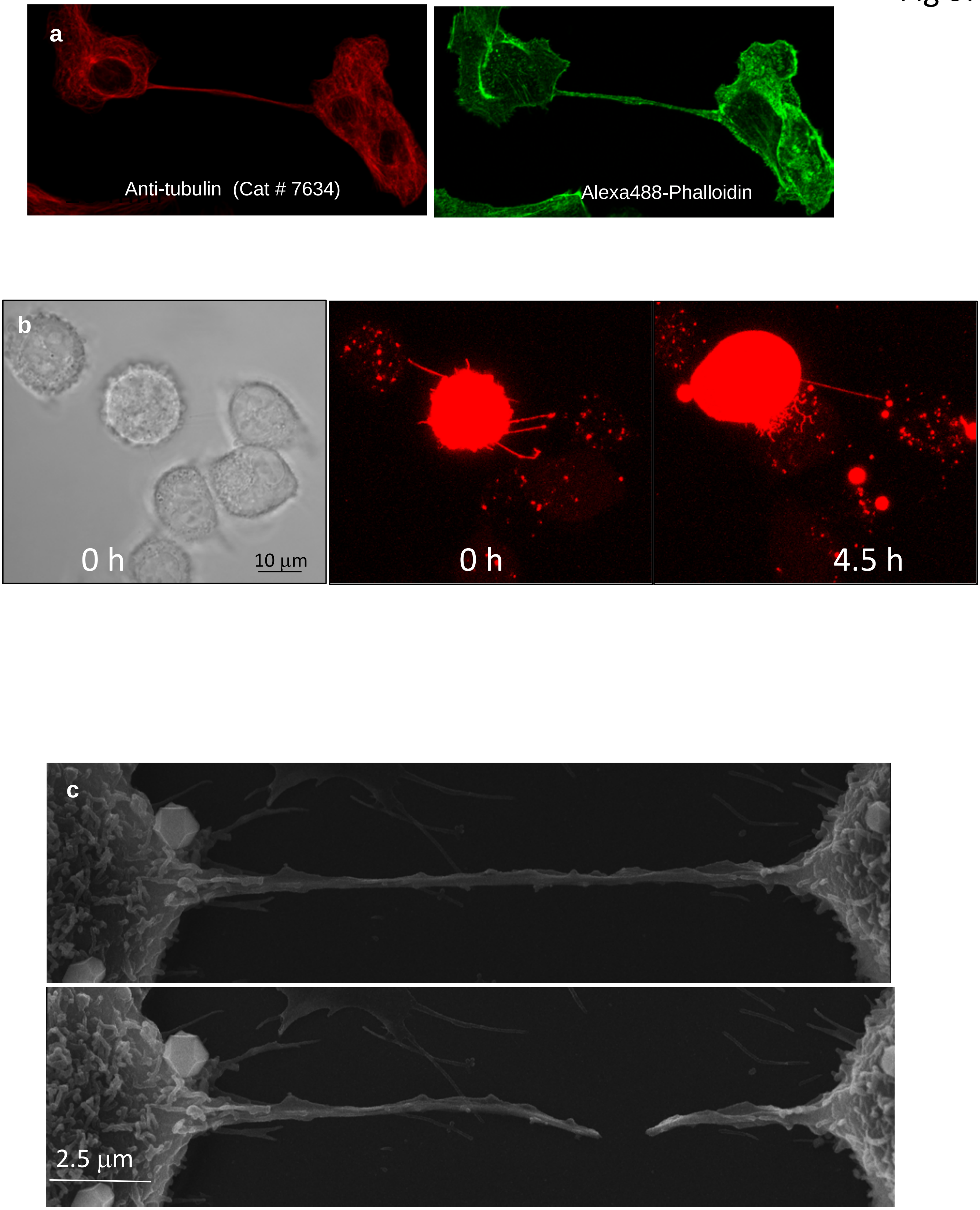
a
Anti-tubulin (Cat # 7634)
Alexa488-Phalloidin
b
0 h
10 µm
0 h
4.5 h
c
2.5 µm

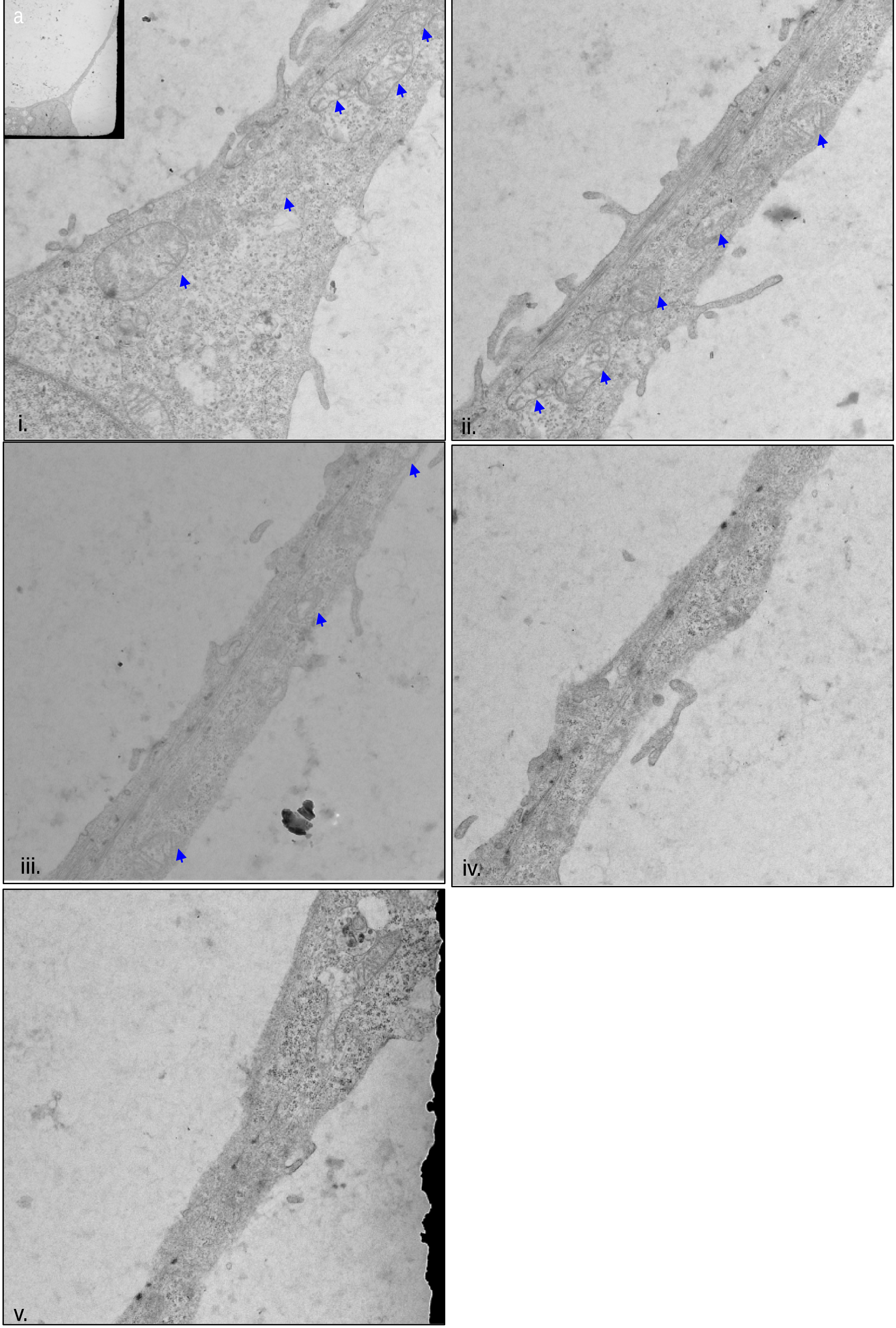

Fig 6.

Fig 7.

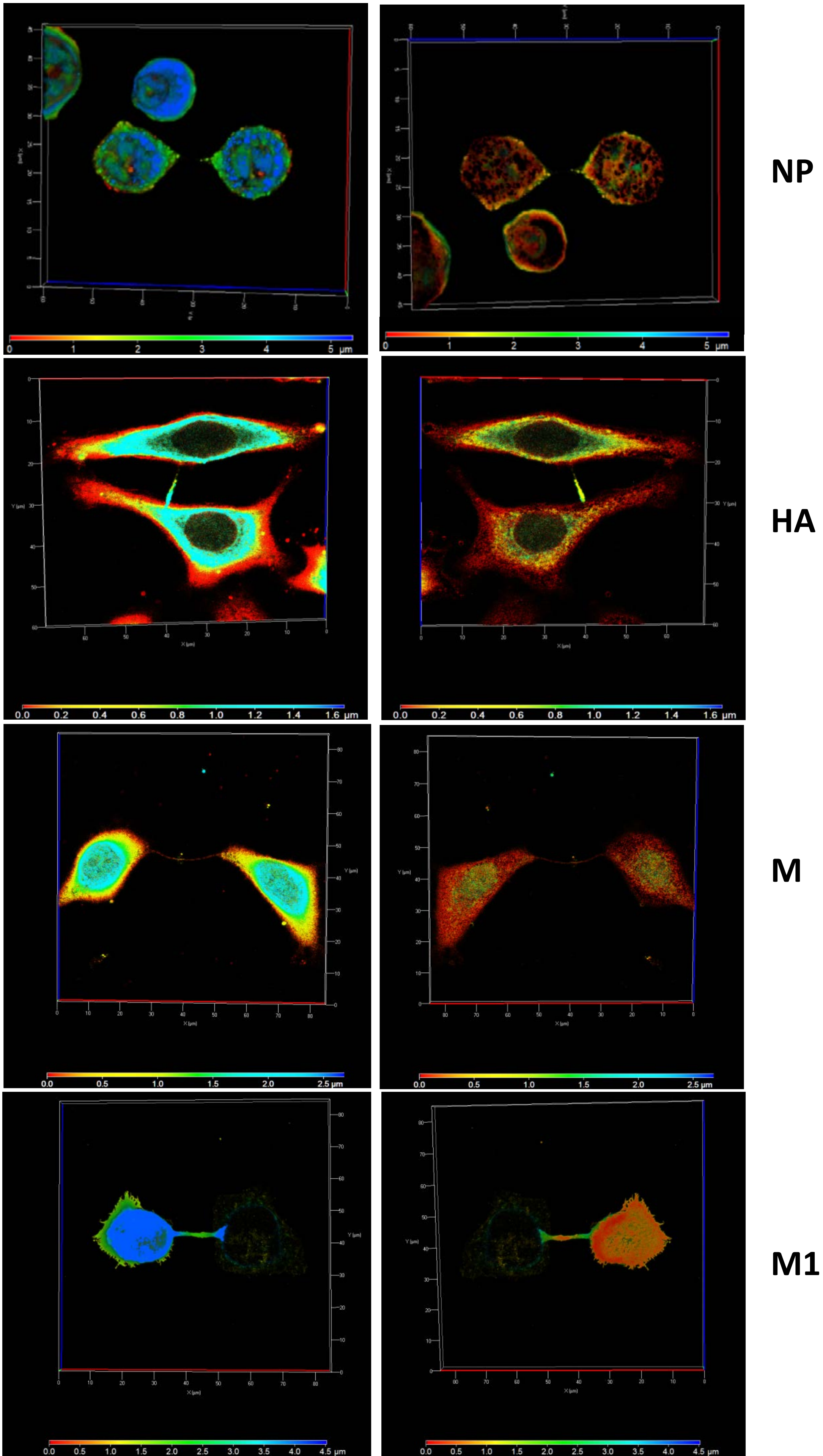
NP
HA
M
M1
0 1 2 3 4 5 µm
0.0 0.2 0.4 0.6 0.8 1.0 1.2 1.4 1.6 µm
0.0 0.5 1.0 1.5 2.0 2.5 µm
0.0 0.5 1.0 1.5 2.0 2.5 3.0 3.5 4.0 4.5 µm

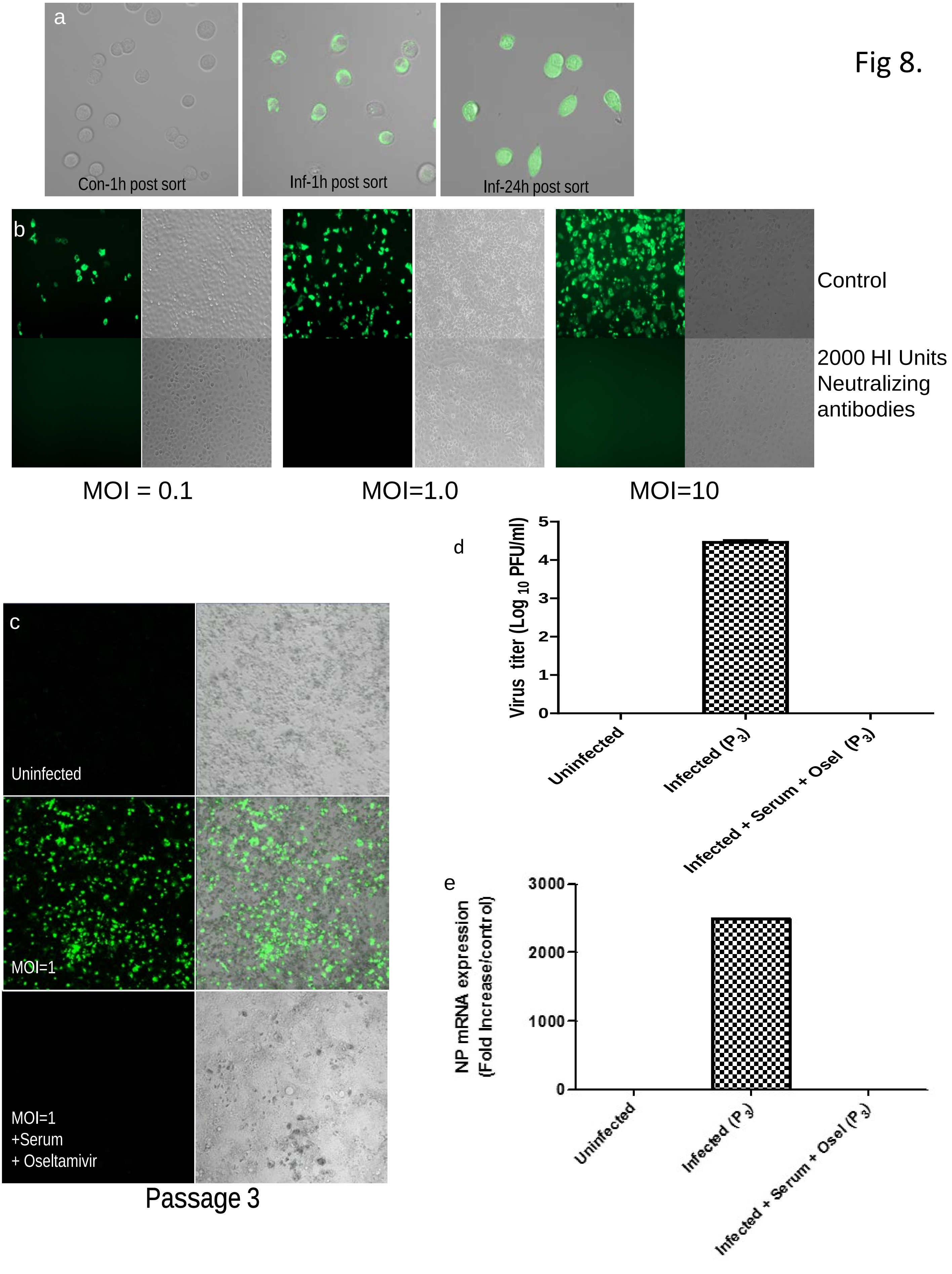
Fig 8.
a
Con-1h post sort
Inf-1h post sort
Inf-24h post sort
b
Control
2000 HI Units Neutralizing antibodies
MOI = 0.1
MOI=1.0
MOI=10
c
Uninfected
MOI=1
MOI=1 +Serum + Oseltamivir
Passage 3
d
Virus titer (Log 10 PFU/ml)
0
1
2
3
4
5
Uninfected
Infected (P3)
Infected + Serum + Osel (P3)
e
NP mRNA expression (Fold Increase/control)
0
1000
2000
3000
Uninfected
Infected (P3)
Infected + Serum + Osel (P3)

Fig9.

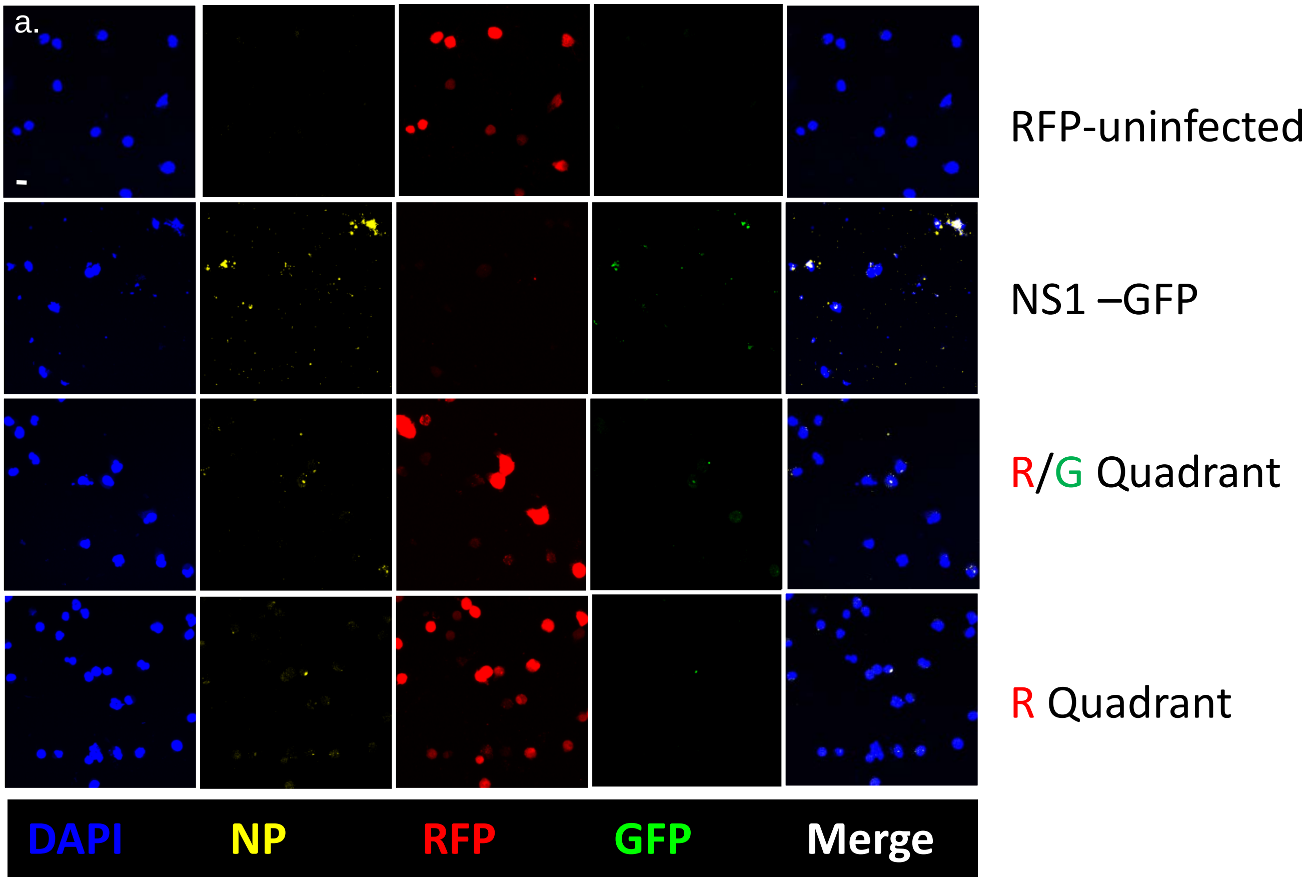

a.
RFP-uninfected
NS1 –GFP
R/G Quadrant
R Quadrant
DAPI
NP
RFP
GFP
Merge

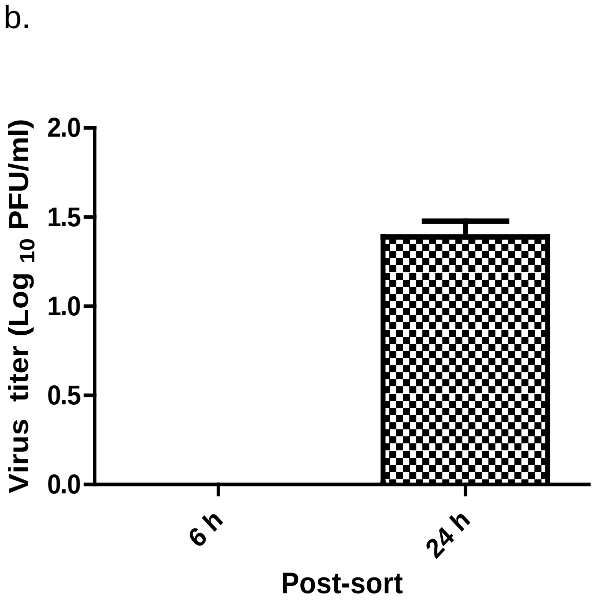

b.
Virus titer (Log 10 PFU/ml)
2.0
1.5
1.0
0.5
0.0
6 h
24 h
Post-sort

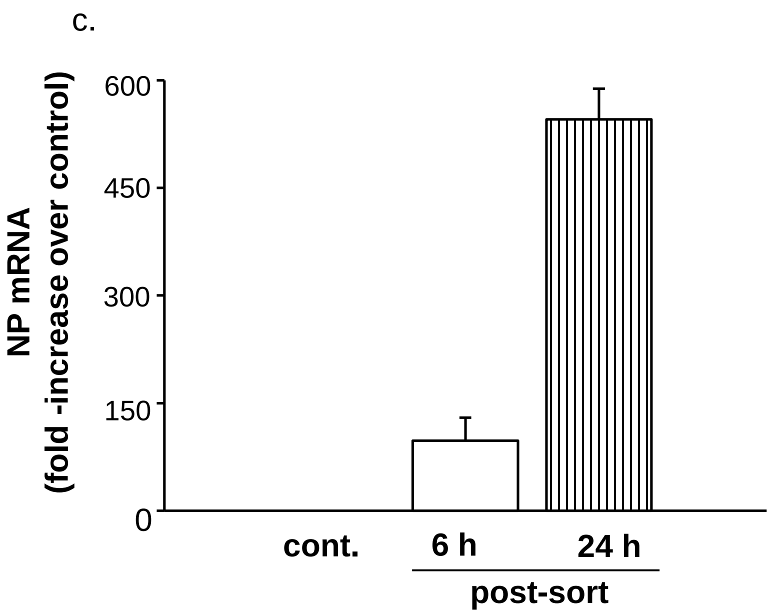

c.
NP mRNA
(fold -increase over control)
600
450
300
150
0
cont.
6 h
24 h
post-sort
